# Serological and Molecular Characterization of Small Ruminant Lentiviruses in Morocco

**DOI:** 10.3390/ani14040550

**Published:** 2024-02-07

**Authors:** Barbara Colitti, Soukaina Daif, Imane Choukri, Daniela Scalas, Anniken Jerre, Ikhlass El Berbri, Ouafaa Fassi Fihri, Sergio Rosati

**Affiliations:** 1 Department of Veterinary Science, University of Turin, Largo Braccini 2, 10095 Grugliasco, TO, Italy; daniela.scalas@unito.it (D.S.); sergio.rosati@unito.it (S.R.); 2 Department of Pathology and Veterinary Public Health, Agronomic and Veterinary Institute Hassan II, BP: 6202, Rabat-Institutes, Rabat 10101, Morocco; soukaina.daif@gmail.com (S.D.); choukrimane7@gmail.com (I.C.); i.elberbri@iav.ac.ma (I.E.B.); o.fassifihri@iav.ac.ma (O.F.F.); 3 Norwegian Veterinary Institute, P.O. Box 64, 1431 Ås, Norway; anniken.jerre@vetinst.no

**Keywords:** small ruminant lentiviruses, divergent strains, serotyping, amplicon sequencing

## Abstract

**Simple Summary:**

The characterization of small ruminant lentiviruses in autochthonous goat and sheep populations, like the Moroccan one, represents an excellent source of information to understand their evolution from domestication to present day and improve the knowledge of the genetic diversity of these viruses. Serological data confirmed a low prevalence likely due to extensive breeding and management practice in this area. However, the low rate of success of routinely used serological tests led us to suppose that divergent strains might have escaped diagnostic tools thanks to mutations’ diagnostic epitopes. This hypothesis is confirmed by next-generation sequencing results, which highlighted the presence of an A subtype carrying a mismatch in the serotyping epitope. Moreover, the circulation of novel B and recombinant A/B subtypes was also revealed.

**Abstract:**

Recent studies that investigated the origins of SRLV strains offered new insights into their distribution among domestic ruminants. The aim of the study was to investigate SRLV circulation in Morocco. A total of 51 farms were selected in different geographical locations and tested by screening and genotyping ELISA. Whole blood was used for DNA extraction and nested gag PCR. The sample size allowed for an estimation of prevalence lower than 20% (CI 95%). Surprisingly, a large proportion of screening-positive samples were not correctly serotyped. Sanger and NGS amplicon sequencing approaches allowed us to obtain new sequences even from difficult-to-amplify samples. The serological data support the evidence of an intrinsic difficulty of SRLV to spread, likely due to management practices. The low rate of success by genotyping ELISA led us to suppose that divergent strains might have escaped from diagnostic tools, as partially confirmed by the evidence of an A subtype carrying a mismatch in serotyping epitope. The sequence analysis revealed the circulation of novel B and recombinant A/B subtypes. This study highlights the importance of monitoring viral sequences and their evolution to develop specific diagnostic tests, particularly in countries where control measures are in place.

## 1. Introduction

Small ruminants have an important economic and social role in Morocco. Farms practicing sheep and goat production represent 98% of livestock farms, with an estimated population of 16 million sheep and 5 million goats. Sheep and goat breeds, mainly represented by old native ones, are well adapted to the local environment, while the limited introduction of French meat breeds occurred in the last decades for terminal crossbreeding [1].

Small ruminant lentivirus (SRLV) infections are distributed worldwide, with few exceptions [2], and are responsible for chronic, slowly progressive diseases known as Maedi Visna in sheep and Arthritis Encephalitis in goats [3,4]. Besides these well documented species-specific diseases, the genetic, antigenic, and biological heterogeneity of SRLV has been well established in the last decades, with important implications in diagnosis and control [5,6]. In particular, the diagnosis of SRLV infections is complicated by the high variability of circulating strains, since no serological tests that are able to discriminate all the possible viral variants are available to date [6]. This limitation was partially overcome by combining the ELISA tests with molecular tools that employed degenerate primers that are able to detect a wide number of subtypes [5,7,8,9,10,11,12,13,14,15,16]. Moreover, in the recent years, novel untargeted molecular techniques have been successfully applied to the study of the genetic diversity and evolution of SRLV strains [17,18].

In this context, the advances in sequencing capacity offer unprecedented opportunities to investigate the host–virus interaction and co-evolution. As considered in previous papers [19,20,21], several lentiviruses have circulated in mammals for millions of years. However, recent studies that investigated the origins of SRLV pandemic strains and their distribution among domestic ruminants demonstrated that the worldwide distribution of the main genotypes occurred relatively recently, in parallel with the increase in the international livestock trade [22]. In particular, phylogenetic reconstruction demonstrated that the worldwide spread of pandemic SRLV-A strains is likely linked to the Karakul sheep export in Europe, Africa, and North America from Central Asia in the early 20th century [23,24]. Moreover, the dissemination of pandemic SRLV-B strains, mainly the B1 subtype, seems to have originated in Central Europe in the mid-20th century, with the export of Saanen dairy goats [24].

In Asia and North Africa, most native sheep are genetically and phenotypically distinct from the sheep found in Europe [24]. In fact, three small ruminant domestication pathways, from West Asia to Africa and Europe, have been demonstrated, and Morocco is located on the Southern Mediterranean one [25]. However, data about SRLV distribution in Northwest Africa are limited, underestimated, and based on a small-scale serological survey that was carried out more than 40 years ago. Thus, no studies documenting the circulating viral genotypes are available, although some incursion may have occurred by the introduction of breeds known to be a carrier of well-characterized European strains [26,27]. The characterization of autochthonous SRLV, if any, in this old population will represent an excellent source of information to understand SRLV evolution from domestication to present day and improve our knowledge of the genetic diversity of this heterogeneous viral population. The aim of the present study was to evaluate the presence and properties of SRLV circulating in local breeds in Morocco based on current knowledge and by using updated approaches and tools.

## 2. Materials and Methods

### 2.1. Sample Selection and Serological Analysis

The samples used in the study belonged to a subset of frozen sera and whole-blood samples collected during a Bluetongue surveillance study carried out between 2019 and 2021 in Morocco [28].

**Figure 1:**
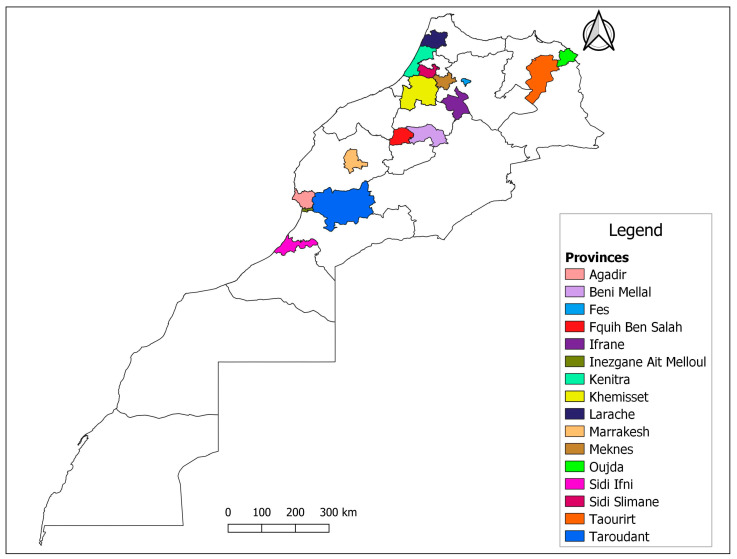
Map of Morocco showing districts sampled during the study.

A total of 755 serum samples (534 sheep and 129 goats, aged > 2 years) were selected from 61 farms (41 sheep and 20 goats) in different geographical locations (Figure 1) and tested for the presence of antibodies against SRLV, using the In3Diagnostic SRLV platform (In3Diagnostic, Torino, Italy).

Briefly, serum samples were antibody tested with the Eradikit™ SRLV screening test (In3Diagnostic, Torino, Italy), and positive and doubt samples were serotyped using the Eradikit SRLV genotyping kit (In3Diagnostic, Torino, Italy), which is normally able to discriminate among genotype A, B, and E in many European countries. For the screening ELISA, the results and sample-to-positive (S/P) ratios were calculated according to the manufacturer’s instructions. For the genotyping, the ELISA results were given as indeterminate; or positive for 1 (A, B, E) or > 1 (e.g., AB) genotype.

As discussed thereafter, a significant subset of samples gave inconclusive results in genotyping ELISA. Based on the sequences obtained with the NGS approach, these latter samples were tested using a modified genotyping ELISA based on the recombinant capsid epitope detected in the field, expressed and purified as described previously [29].

Briefly, the sequence encoding the variant capsid epitope from Morocco was PCR amplified and cloned into the PGex-6P prokaryotic vector. The gene subunit was expressed, upon induction, in E. coli BL21 cells, in frame with GST tag and purified from cell lysate, using the glutathione Sepharose 4B affinity matrix. The recombinant capsid antigen was used in the ELISA format to evaluate the reactivity of this subset of samples.

### 2.2. DNA Extraction and SRLV Proviral Amplification

Genomic DNA was extracted from paired whole-blood samples with a DNeasy Blood and Tissue Kit (Qiagen, Hilden, Germany), following manufacturer’s instructions, and quantified with Nanodrop 2000 Spectrophotometer (Thermo Scientific, Madison, WI, USA). DNA was used to amplify about 800 bp long sequences of SRLV *gag–pol* genes, using a previously reported nested PCR [30]. PCR products were visualized in 1.5% agarose gel, and positive samples were purified using a Gel and PCR purification kit (Macherey-Nagel, Hœrdt, France), following manufacturer’s instructions. Purified samples were submitted to an external laboratory for Sanger sequencing (BMR Genomics, Padova, Italy). Although the method allowed the use of up to 1 ug of DNA from buffy coat, the amount of DNA extracted from frozen blood was much lower, with an average of 20.9 ng/µL.

### 2.3. Amplicon Sequencing

Samples with discordant serology/PCR results or with multiple or very tiny bands on the agarose gel, making them not feasible for Sanger sequencing, were submitted to NGS amplicon sequencing as follows.

Briefly, PCR reactions were purified using a Gel and PCR purification kit (Macherey-Nagel). PCR amplified DNA was quantified with a fluorimetric method, Qubit double-strand DNA (dsDNA) High Sensitivity assay kit on a Qubit 3.0 instrument (Life Technologies, Leominster, MA, USA) and subjected to tagmentation, amplification, and indexing, using the Illumina DNA prep Kit and IDT for Illumina UD indexes (Illumina, San Diego, CA, USA), according to the manufacturer’s protocol. Libraries were then normalized to a 4 nM concentration, pooled, and denatured with 0.2 N sodium acetate. The 12.5 pM paired-end library was spiked with 5% PhiX control and sequenced on an Illumina Miseq platform, using a V2-500 cycles chemistry. 

### 2.4. Data Analysis

Sanger sequencing and NGS data were analyzed using Geneious Prime v. 2023.2.1 software as previously reported [17].

Briefly, Illumina amplicon sequencing reads were checked for sequence quality by using FastQC [31] and then aligned to known reference genomes to identify and confirm the viral genotype. The reads were further aligned to the consensus sequence obtained after the first step in order to confirm the genome sequence. A de novo approach was also performed using Velvet software ver. 1.2.10 [32], and the obtained contigs were compared to the consensus sequence derived from resequencing.

The phylogenetic analysis was based on the partial *gag–pol* genes previously described by Shah [22]. The partial *gag–pol* fragment of 16 Moroccan sequences was aligned with 71 reference strains retrieved from GenBank, using ClustalW [33], which is included within the software Geneious Prime ver. 2023.2.1.

The partial gag gene was used to depict phylogenetic relationships between the newly characterized and the reference strains using a Bayesian approach implemented in MrBayes package [34] with the GTR + G + I substitution model (bootstrap values of 1000 replicates). The tree topology was also confirmed with the maximum likelihood inference method with the Tamura-Nei model (bootstrap values of 1000 replicates). 

To assess amino acid positions under selection pressure, the alignment of the 16 Moroccan gag sequences was used to estimate substitution rates at non-synonymous (dN) and synonymous (dS) sites, and their ratio (dN/dS), using the Single likelihood ancestor counting (SLAC) method implemented in Datamonkey webserver [35]. Positions with a dN/dS ratio > 1 were considered to be under positive selection.

The presence of recombinant strains was investigated using RDP4 v. 4.101 and SimPlot v.3.5.1 software [36,37]. 

Nucleotide sequences were deposited in GenBank (Accession numbers from OR682453 to OR682468).

## 3. Results

### 3.1. Serology

In total, 51 samples out of 755 (7 goats and 44 sheep) gave positive or doubtful results in the screening ELISA. In particular, 32 samples from sheep and 1 from goats gave a clear positive result, while 6 goats and 12 sheep gave a doubt result in the screening assay. Interestingly, only 20 out of 51 samples that resulted in positive or doubtful to the screening ELISA test were correctly serotyped (Table 1). The prevalence of antibody-positive sheep and goat was 5.3% and 0.7%, respectively. The sample size allows for an estimation of prevalence lower than 20% (CI 95%) in 41 sheep and 20 goat flocks.

Interestingly, 31 samples gave positive or doubt results in the screening ELISA but tested indeterminate or negative in the genotyping ELISA (Table 1).

The amino acid alignment showed the presence of mismatches in the sequence encoding the immunodominant capsid epitope used for serotyping purpose in samples Mor08, Mor018, Mor020, Mor021, and Mor022 (Table 2). 

**Figure 2:**
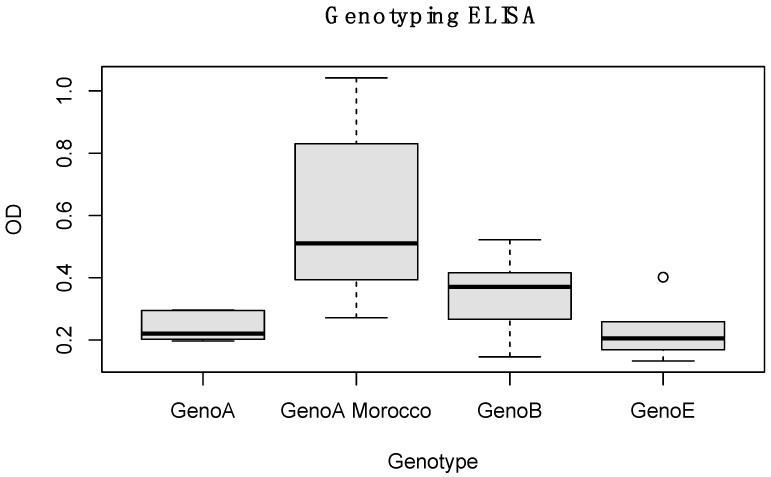
Boxplot showing the reactivity of a subset of sera, previously detected as negative to all genotypes in the Eradikit genotyping ELISA (Geno A, GenoB and GenoE), to the newly developed ELISA, based on the genotype A variant capsid epitope (GenoA Morocco).

Considering these findings, the 31 samples, which previously resulted in being negative with the Eradikit genotyping ELISA, were tested with the newly developed indirect ELISA based on the genotype A variant capsid epitope. Thus, 17 out of 31 samples turned out to be positive and were correctly serotyped, as shown in Figure 2.

### 3.2. Sequencing

Genomic DNA was extracted from fifty-one serology-positive or -doubtful samples, with an average concentration of 20.9 ng/uL (95% CI, 15.1–26.7), and was subjected to the nested *gag-800* PCR. Only four samples gave a clear band of the expected size of 800 bp and were submitted to Sanger sequencing. Among the remaining samples, 12 gave tiny or multiple bands or smear in the agarose gel and underwent an NGS approach. All the 12 samples gave a positive result in amplicon sequencing, with an average depth of coverage that spanned from 1234 to 226,000×, having 3633–1,224,740 aligned reads, representing ~100% amplicon coverage. Interestingly, even if only the second nested PCR (800 bp) was purified, the sequence retrieved with the NGS approach almost covered the 1300 bp of the first PCR for 11 out of 12 samples. 

The phylogenetic analysis was based on the alignment of 779 nucleotides of the 16 sequences (3 from goats and 13 from sheep; 4 obtained with Sanger sequencing and 12 with amplicon sequencing) from Morocco and 71 reference strains from groups A and B and rooted to group E. 

**Figure 3:**
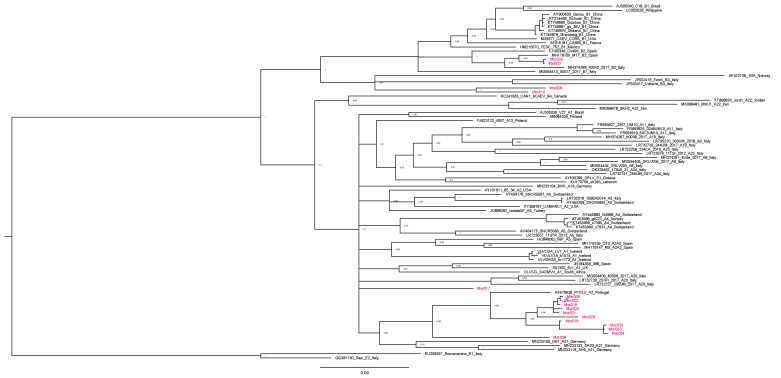
Phylogenetic tree based on the alignment of 779 bp of gag sequence of reference and newly characterized Moroccan strains (in red). The accession number, name, subtype, and country of detection are reported on the label for each reference sequence. Bootstrap values are shown above the respective branches. The bar indicates the amount of evolution along the horizontal branches in substitutions per site.

The sequence analysis revealed the presence of both A and B genotypes (Figure 3 and Table 3).

The great part of ovine sequences segregated with group A strains (Figure 3); this result is consistent with previous, though dated, reports [26,27].

Interestingly, eleven samples showed a large difference with other reference sequences (at least around 22%) belonging to the same monophyletic clade, together with A2 P1OLV Portuguese subtype, confirming the very high heterogeneity of MVV-like viral strains. Interestingly, one sequence, Mor0017, showed a mismatch in the capsid epitope encoding sequence (Table 2) and clustered into the A1/A2 groups, even if supported by a lower bootstrap value that did not allow us to attribute the sequence to a subtype. Interestingly, this finding is similar to what has been reported in a previous study in Finland [13]. 

Considering the classification based on nucleotide difference reported by Shah et al. [22], two ovine sequences (Mor009 and Mor010) showed a genetic diversity ranging from 17.8 to 21.6% with reference B strains (pairwise identity of 82% and 81.6% for Mor009 and 79.3% and 78.4% for Mor010 with B1 Cork and B3 Fonni reference strains, respectively—Acc. Nos. M33677 and JF502416), suggesting the presence of a new B6 subtype.

Interestingly, NGS data also confirmed mismatches in the aligning primer region for some sequences retrieved in this study, partially explaining the low rate of success of the nested PCR. 

Moreover, a number of recombination analyses were performed on all the SRLV sequences from Morocco, using the RDP4 v.4.101 software. Interestingly, similar recombination signals were detected with all methods included in the RDP4 package and further confirmed with Simplot v. 3.5.1 software for one sequence (Mor010).

**Figure 4:**
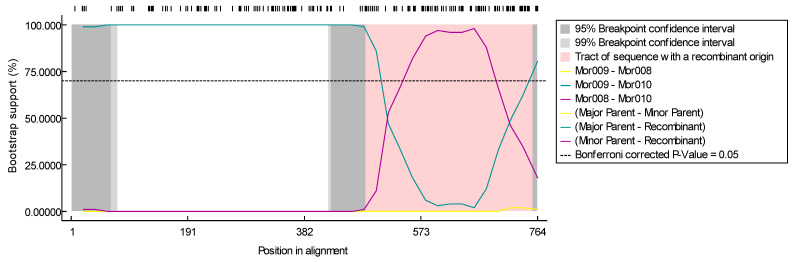
Recombination analysis using RDP4 v.4.101 software. Boot-scanning plot of the *gag* gene of Mor010 sequence against the gag gene derived from strains Mor009 and Mor008 is depicted, with region of recombinant origin highlighted in pink.

According to the RDP analysis, the Mor010 sequence was derived from a recombination event between B genotype sequence (Mor009) and A subtype (Mor008/Mor017) sequence. The correct breakpoints of this recombination event were mapped in the consensus sequence created in the alignment of the three sequences and analyzed by boot-scanning, using the Simplot software. Breakpoints were both located within the *gag* gene subunit encoding for major capsid antigen (Figure 4). Consistent with the recombination and phylogenetic analyses, the Mor010 sequence appeared to be related to both the A and B genotype strains since they were equidistant from both groups. 

**Figure 5:**
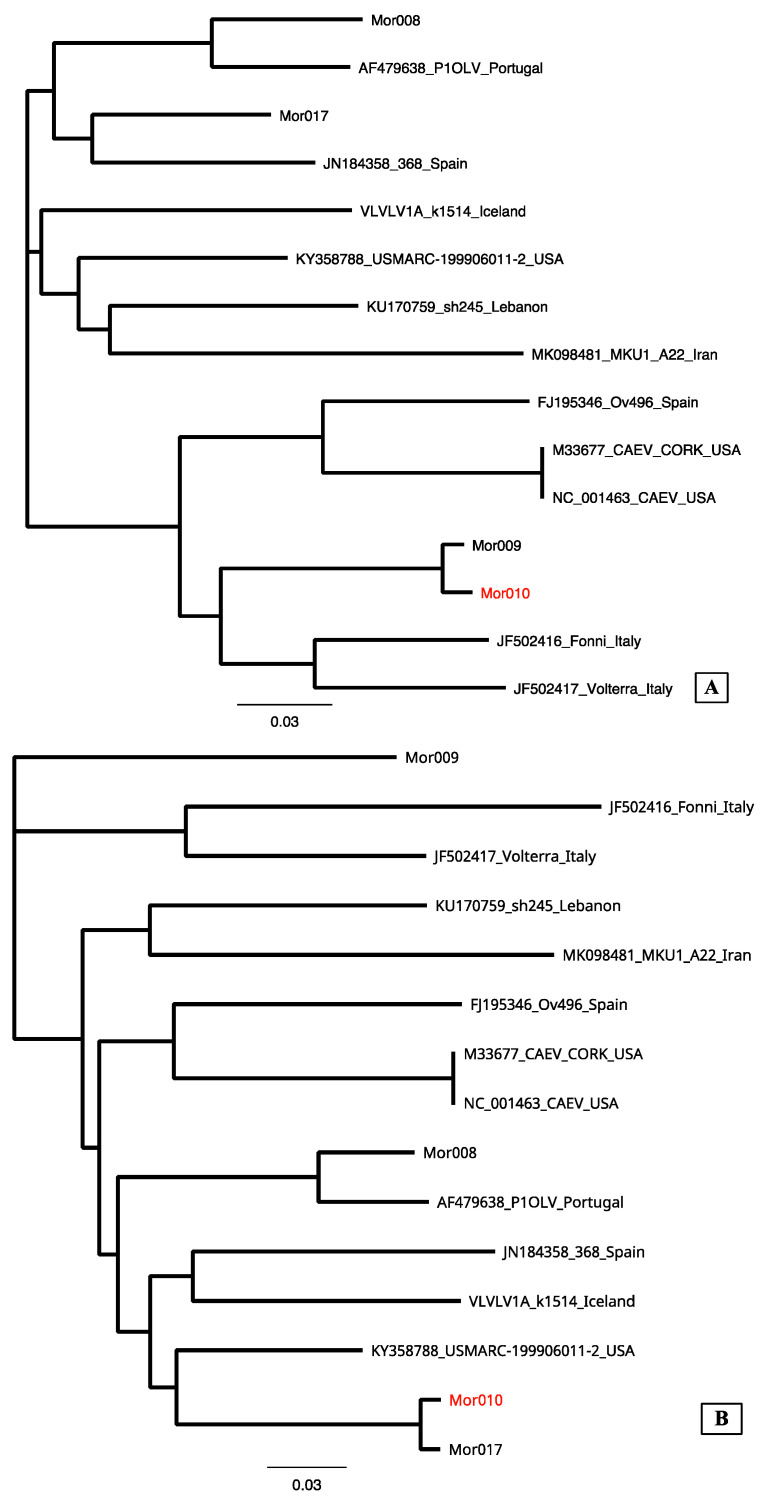
Neighbor-joining trees showing phylogenetic relationships of the recombinant strain Mor010 (in red) and reference strains prior to the breakpoint, nucleotides 1 to 500 (**A**); and within the recombined gene segment, nucleotides 501–764 (**B**).

In order to confirm this finding, neighbor-joining phylogenetic analyses were performed on two sub-fragments from a 764 bp alignment of gag sequence (position 1 to 500—matrix; and 501 to 764—capsid segment) of Mor010 and major reference strains. The analysis of the phylogenetic trees supported the recombination results since the Mor010 sample clustered closely with B6 subtypes in the matrix segment and with A2 subtypes in the capsid segment (Figure 5). This clustering pattern supports the evidence that the Mor010 strain is a mosaic sequence derived from a recombination event between parental strains of group A2 and group B6.

Finally, in order to estimate the selection along the coding region of the newly characterized sequences, the average substitution rates at non-synonymous (dN) and synonymous (dS) sites and their ratio (dN/dS) were determined. An average ratio of 0.0940, with a *p*-value threshold of 0.1, suggested the existence of a purifying selection that acts to remove deleterious mutations from this coding region.

## 4. Discussion

Small ruminant lentivirus infections and epidemiology have been widely studied in Mediterranean countries since early eighties, and the data available to date suggest that genetic and antigenic heterogeneity reflect the variability of the error-prone transcriptase on the one hand and animal migration and, more recently, animal trade on the other [24,38,39,40]. Different incursions of SRLV occurred in the past, such as for the introduction of genotype E in Sardinian goats, genotype C in Norwegian goats, or the more recent spread of different genotype B subtypes in sheep (like B2 or B3 within the Mediterranean basin) [3,38,41]. One advantage of SRLV persistence is the tendency to become endemic in intensive rearing systems, like in the dairy goat industry. Therefore, it is not rare, without implementation of control measures, to detect a high prevalence of infection in such breeds. However, the introduction of SRLV in naive animal populations in conditions that are believed to be not well suited for SRLV persistence, such as more extensive rearing systems and/or climate conditions, is less studied. 

The small ruminant population in Morocco is believed to have originated about 7000 years before present, domesticated outside Africa, and entered via the Northwest African migration route [42]. The full characterization of autochthonous SRLV, if any, in this old population, will represent an excellent source of information to understand SRLV evolution after domestication and may represent a useful model to evaluate the ability, if any, of such a group of viruses to persist, even at low prevalence, in the small ruminant flocks, improving our knowledge of the genetic diversity of this heterogeneous viral population.

With this aim, we first conducted a serological survey by searching for the presence of antibodies in a subset of samples covering different regions, breeds, and rearing systems. Based on our data, the overall prevalence was around 5% in sheep and 0.7% in goats. In about 50% of the selected flocks, no positive sera were detected, and based on sample size, an estimation of prevalence lower than 20% (CI 95%) could be realistic in those flocks. Taken together, these data suggest that, with very few exceptions, SRLV infection occurs in Morocco at a low level of endemicity. However, considering that the screening test was based on recombinant antigens derived from European viral sequences, we cannot exclude that our antibody test may have suffered from poor sensitivity due to antigenic variation in local strains, as previously suggested [43,44]. This hypothesis is further supported by the limited success in serotyping the doubtful and positive samples. The genotyping test, indeed, displays a normal success rate greater than 80% in different European countries, while it did not reach 40% in this study. The genetic characterization of local strains was therefore imperative to clarify this assumption. Unfortunately, only paired frozen blood samples were available in the panel and, even if an adapted protocol for DNA extraction was available, an average DNA amount of 20 ng in each PCR reaction was amplified (min 6 ng, max 38 ng), while up to 500 ng is suggested to reach the highest sensitivity [30]. The limited number of nested PCR-positive samples may reflect the low DNA concentration obtained from whole blood, the low viral load, the presence of mismatches in the primer binding sites, or a combination of these factors. Thus, we still could not exclude, at this stage of the study, the idea that highly divergent strains might have escaped from diagnostic tools. To cover this gap, we analyzed a discrete number of PCR-negative/antibody doubt or positive samples, by NGS amplicon sequencing, combining the first and the second PCR reactions. The “blind approach” represented by NGS enabled us to overcome both the issue of the low concentrations of starting material and the low sensitivity of PCR primers. It allowed us to retrieve the target sequence even in those samples in which a clear band in the agarose gel was not visible.

Surprisingly, an additional 12 SRLV sequences were obtained, and although coverage was rather low, the full 1.3 Kb amplicon was assembled for most samples, leading us to suppose that mismatches at the primer binding sites mainly affected the second PCR run. This finding, once more, confirms the difficulty in developing a unique molecular assay that is able to universally detect all the possible SRLV subtypes, as previously reported in different attempts [6]. 

Ovine viral sequences (13/16) reflect multiple incursions of SRLV from neighboring countries at different times. Most of the genotype A sequences clustered with strain P1OLV, a subgroup A2 characterized in Portugal, and one sequence clustered with A1 strains and the Spanish isolate 368, recently associated with an outbreak of neurological disease [45]. Interestingly, this sequence, Mor017, showed a mismatch in the capsid epitope encoding sequence, also identified in a Finnish sequence previously published [13] and, thus, in a country where a compulsory program for SRLV has been implemented since 2001. This finding, together with the high heterogeneity found in A-subtype sequences retrieved in the study, in the absence of selective pressure due to control measures that can justify the presence of escape mutants in antigens of diagnostic relevance, highlighted how the variability into the A genotype may be even greater than expected.

Regarding genotype B, two subtypes were detected: a B2 subtype, which was more recently introduced from South European countries (Spain, France, or Italy); and a putative novel subtype, tentatively assigned to B6, found in a single sheep flock in the Taourit region. This single sequence may reflect a more ancient introduction, being more related (81%) to subtype B3, characterized in Sardinian and Turkish sheep. Finally, a potential A/B recombination event was detected in a Sardi sheep in the same Taourit region. Among goat sequences, A2 and B2 subtypes were identified.

Moreover, considering the selection pressure for this region, the dN/dS ratio, which estimates the relative rate of selection versus neutral changes across protein-coding genes [46], confirmed the impact of purifying selection on this region as previously reported [16,47,48].

To further evaluate the low success rate of serotyping ELISA, we analyzed the major capsid epitope and found that most of the A2 subtype sequences harbored a single AA mutation (P/A), which dramatically affected the sensitivity of the method. By introducing the recombinant peptide corresponding to this variant, the correct reactivity was readily established. It is noteworthy that this viral cluster was selected as a diagnostic escape mutant in the absence of a diagnostic pressure, and its evolutionary advantage, if any, to date remains unknown.

## 5. Conclusions

In conclusion, the serological data reported a low prevalence of infection in Morocco with an intrinsic difficulty of SRLV to spread likely due to the farming system, based on extensive breeding with isolated flocks. Despite this low prevalence and the absence of control measures, a great variability in circulating strains was recorded, stressing once more the importance of monitoring the evolution of SRLVs in particular in immunodominant diagnostic antigens and in those countries where control programs have been implemented for several years.

## Figures and Tables

**Table 1:** Data of the 61 flocks tested.

Region	Species	Number of Flocks Tested	Number of Animals	Number of Tested Animals	Screening Positive	Screening Doubt	Genotyping A	Genotyping B	Genotyping E	Genotyping Indeterminate	Genotyping Negative
Agadir	Sheep	9	1896	151	3	6	0	3	0	2	4
	Goat	7	358	86	1	6	0	2	2	1	2
Marrachech	Sheep	8	515	91	1	2	0	1	0	2	0
	Goat	2	22	7	0	0	-	-	-	-	-
Taourirt	Sheep	2	700	58	8	1	1	1	0	4	3
	Goat	2	20	9	0	0	0	0	0	0	0
Kenitra	Sheep	3	140	65	0	0	-	-	-	-	-
Laraache	Sheep	3	210	43	7	0	0	0	1	3	3
Kcer Lkbir	Sheep	2	100	38	7	0	3	0	3	0	1
Fes	Sheep	1	50	18	2	0	0	0	0	1	1
Ifrane	Sheep	2	110	29	0	1	0	0	0	1	0
Fquih Ben Salem	Sheep	2	220	20	1	0	0	0	0	1	0
	Goat	2	40	12	0	-	-	-	-	-	-
Rommani	Sheep	4	74	48	3	0	1	0	0	2	0
	Goat	1	5	1	0	-	-	-	-	-	-
Meknes	Sheep	1	75	9	0	-	-	-	-	-	-
Oujda	Sheep	3	140	25	0	2	0	2	0	0	0
	Goat	1	2	2	0	-	-	-	-	-	-
Sidi Ifni	Goat	3	87	21	0	-	-	-	-	-	-
Benimellal	Goat	1	17	10	0	-	-	-	-	-	-
Sidi Slimane	Goat	1	15	5	0	-	-	-	-	-	-
Berkane	Sheep	1	20	7	0	-	-	-	-	-	-

**Table 2:** Alignment of the capsid immunodominant epitope of the newly characterized Moroccan sequences, using reference strains belonging to different subtypes and countries.

Sequence	Subtype	Alignment
VLVLV1A_k1514_Iceland	A1	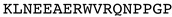
Mor019		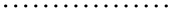
Mor023		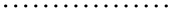
Mor026		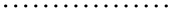
Mor033		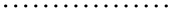
Mor034		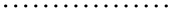
AY101611_85-34_USA	A2	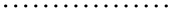
AY454175_SNCR5560_Switzerland	A5	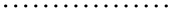
AY454176_5561_Switzerland	A3	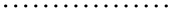
AY454208_5692_Switzerland	A7	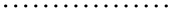
MH374287_It0038_2017_Italy	A19	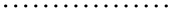
MH374291_ItVda_2017_Italy	A8	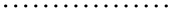
MK098481_MKU1_Iran	A22	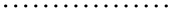
MG554409_It0009_2017_Italy	A20	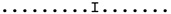
Mor017		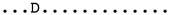
AM084209_Finland	A	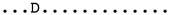
Mor008		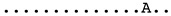
Mor018		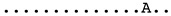
Mor020		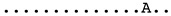
Mor021		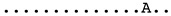
Mor022		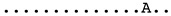
AY445885_G4668 _Switzerland	A4	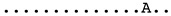
AF479638_P1OLV_Portugal	A2	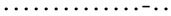
M33677_CAEV_CORK_USA	B1	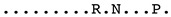
Mor038		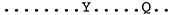
Mor009		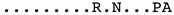
Mor010		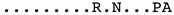
JF502416_Fonni_Italy	B3	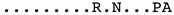
GU120138_Shanxi _China	B1	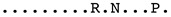
KT214469_Sichuan_China	B1	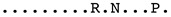
HM210570_FESC-752_Spain	B1	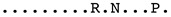
LC002526_Philippine	B	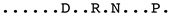
Mor035		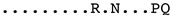
Mor037		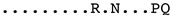
FJ195346_Ov496_Spain	B2	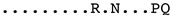
AF015181_CA680_France	B1	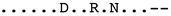
EU293537_Roccaverano _Italy	E1	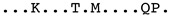
GQ381130_Seui _Italy	E2	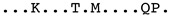
AF322109_1GA_Norway	C	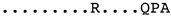

**Table 3:** Serology and sequencing data results.

Sample	Region	Specie	ELISA Screening	ELISA Genotyping	PCR	Subtype
Mor008	Taourirt	Ovine	Doubt	Indeterminate	Positive	A2
Mor009	Taourirt	Ovine	Positive	B	Positive	B6
Mor010	Taourirt	Ovine	Doubt	Indeterminate	Positive	B6
Mor017	Agadir	Caprine	Positive	Indeterminate	Positive	A
Mor018	Agadir	Caprine	Doubt	Indeterminate	Positive	A2
Mor019	Rommani	Ovine	Doubt	A	Doubt	A2
Mor020	Laraache	Ovine	Doubt	Negative	Doubt	A2
Mor021	Laraache	Ovine	Doubt	Negative	Doubt	A2
Mor022	Laraache	Ovine	Doubt	Negative	Doubt	A2
Mor023	Kcer Lkbir	Ovine	Positive	E	Doubt	A2
Mor026	Fes	Ovine	Positive	Negative	Positive	A2
Mor033	Kcer Lkbir	Ovine	Positive	Negative	Positive	A2
Mor034	Kcer Lkbir	Ovine	Positive	A	Positive	A2
Mor035	Agadir	Caprine	Negative	B	Doubt	B2
Mor037	Rommani	Ovine	Doubt	Indeterminate	Positive	B2
Mor038	Taourirt	Ovine	Positive	E	Positive	A2

## Data Availability

Sequencing data produced in this paper are available in GenBank with Accession numbers from OR682453 to OR682468.
